# Spine Enlargement of Pyramidal Tract-Type Neurons in the Motor Cortex of a Rat Model of Levodopa-Induced Dyskinesia

**DOI:** 10.3389/fnins.2017.00206

**Published:** 2017-04-13

**Authors:** Tatsuya Ueno, Haruo Nishijima, Shinya Ueno, Masahiko Tomiyama

**Affiliations:** ^1^Department of Neurology, Aomori Prefectural Central HospitalAomori, Japan; ^2^Department of Neurophysiology, Hirosaki University Graduate School of MedicineHirosaki, Japan

**Keywords:** Parkinson's disease, dyskinesia, levodopa, motor cortex, dendritic spines, plasticity, 6-hydroxydopamine, pyramidal neuron

## Abstract

Growing evidence suggests that abnormal synaptic plasticity of cortical neurons underlies levodopa-induced dyskinesia (LID) in Parkinson's disease (PD). Spine morphology reflects synaptic plasticity resulting from glutamatergic transmission. We previously reported that enlargement of the dendritic spines of intratelencephalic-type (IT) neurons in the primary motor cortex (M1) is linked to the development of LID. However, the relevance of another M1 neuron type, pyramidal-tract (PT) neurons, to LID remains unknown. We examined the morphological changes of the dendritic spines of M1 PT neurons in a rat model of LID. We quantified the density and size of these spines in 6-hydroxydopamine-lesioned rats (a model of PD), 6-hydroxydopamine-lesioned rats chronically treated with levodopa (a model of LID), and control rats chronically treated with levodopa. Dopaminergic denervation alone had no effect on spine density and head area. However, the LID model showed significant increases in the density and spine head area and the development of dyskinetic movements. In contrast, levodopa treatment of normal rats increased spine density alone. Although, chronic levodopa treatment increases PT neuron spine density, with or without dopaminergic denervation, enlargement of PT neuron spines appears to be a specific feature of LID. This finding suggests that PT neurons become hyperexcited in the LID model, in parallel with the enlargement of spines. Thus, spine enlargement, and the resultant hyperexcitability of PT pyramidal neurons, in the M1 cortex might contribute to abnormal cortical neuronal plasticity in LID.

## Introduction

Parkinson's disease (PD) is characterized by the loss of dopaminergic neurons in the substantia nigra of the midbrain, resulting in bradykinesia, muscular rigidity, rest tremor, and postural instability (Gibb and Lees, 1988). The most effective treatment for PD is oral administration of the dopamine precursor, L-3,4-dihydroxyphenylalanine (levodopa) (Olanow et al., 2006). However, long-term treatment with levodopa induces a variety of abnormal involuntary movements, termed levodopa-induced dyskinesia (LID), which represent a major treatment limitation and reduce the quality of life of PD patients (Olanow et al., 2006).

The emergence of these abnormal involuntary movements is associated with altered corticostriatal synaptic plasticity (Picconi et al., 2003). Electrophysiological recordings performed in corticostriatal slices of 6-hydroxydopamine (6-OHDA) lesioned rats with LID have shown that depotentiation at corticostriatal synapses to direct pathway striatal projection neurons (dSPN) is lost after the induction of long-term potentiation (LTP) (Shen et al., 2015). Depotentiation reverses synaptic strength from the potentiated state to pre-LTP levels, which is implicated in the mechanisms of physiological “forgetting” (Picconi et al., 2003). Consequently, the absence of depotentiation may result in the storage of unessential motor information, suggesting a key neurophysiological feature of LID (Picconi et al., 2003). Synapse strength can be determined by alteration of spine volume, or enlargement or shrinkage of spines (Kasai et al., 2010). Indeed, in a rat model of LID, we showed that dSPN dendritic spines became enlarged, suggesting supersensitivity of the corticostriatal excitatory synapses of dSPNs (Nishijima et al., 2014).

Dopaminergic signaling within the primary motor cortex (M1) is necessary for normal motor skill learning and synaptic plasticity (Molina-Luna et al., 2009). Dopaminergic projections to M1 arise from the ventral tegmental area (Hosp et al., 2011), in which neurons are also lost in PD patients (Uhl et al., 1985). Thus, progressive degeneration of dopaminergic neurons in the ventral tegmental area leads to decreased endogenous dopamine in the cortex, which affects synaptic plasticity in the M1 (Huang et al., 2011). In human studies, M1 plasticity is investigated using motor-evoked potential amplitudes elicited by transcranial magnetic stimulation (Huang et al., 2011). Using this method, PD patients with LID exhibit a lack of depotentiation-like cortical plasticity (Huang et al., 2011). This suggests that unessential motor information accounting for LID is stored in both in the striatum and the M1 (Picconi et al., 2003; Huang et al., 2011).

In rodents, corticostriatal neurons in the motor cortex are categorized into two main types: intratelencephalic (IT) and pyramidal tract (PT) neurons (Reiner et al., 2010). It has been demonstrated that IT neurons preferentially innervate dSPNs in the ipsilateral and contralateral striatum, whereas PT neurons preferentially innervate SPNs of the indirect pathway (iSPN) in the ipsilateral striatum, and send axons to the brainstem via the pyramidal tract (Reiner et al., 2010). It has been reported that dSPNs appear to play an important role in the development of LID (Picconi et al., 2003; Shen et al., 2015). In a previous study, we found enlargement of IT neuron spines in a LID model rat and proposed that IT neurons in the M1 may store abnormal information resulting in LID (Ueno et al., 2014). Furthermore, IT neurons in the M1 of the LID rat model displayed increased amplitudes of miniature excitatory postsynaptic currents (Ueno et al., 2014). These data suggest that IT neurons in dyskinesia-primed animals acquire supersensitivity to excitatory stimuli (Ueno et al., 2014).

However, it has been demonstrated that dSPNs and iSPNs are innervated by both PT and IT neurons (Kress et al., 2013; Deng et al., 2015). Thus, the preferential innervation from IT and PT neurons to SPNs remains controversial (Deng et al., 2015). Thus, it is conceivable that PT neurons also play an important role in the development of LID. Therefore, we investigated the density and size of PT neuron spines in the M1 in rat models of PD and LID.

## Materials and methods

### Experimental animals

Male Wistar rats (Japan Clea Co. Ltd., Tokyo, Japan) were housed in a temperature-controlled room (~25°C) with a 12-h day/night cycle, with free access to food and water. This study was conducted in accordance with the guidelines for animal research issued by the Physiological Society of Japan and by Hirosaki University School of Medicine with the approval of Hirosaki University Animal Experimentation Committee.

### Creation of rat models

**Figure 1:**
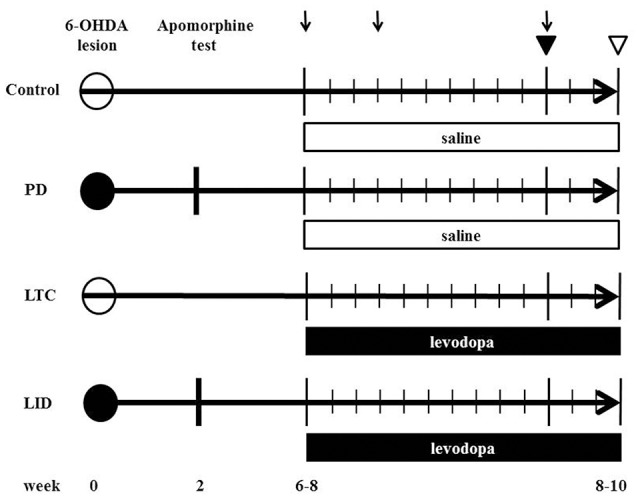
**Time chart and experimental design of the study**. We injected 6-hydroxydopamine (6-OHDA) or saline into the medial forebrain bundle to induce hemiparkinsonian (levodopa-induced dyskinesia model [LID]; Parkinson's disease model [PD]) or sham-operated rats (levodopa-treated control [LTC]; Control), respectively. The number indicates the weeks post 6-OHDA lesion. At 2 weeks after 6-OHDA, dopaminergic denervation was confirmed by apomorphine test. LID and LTC rats received daily levodopa treatment for 2 weeks, while PD, and Control rats received daily saline for 2 weeks. Closed and open circles indicate 6-OHDA and saline injection, respectively. Arrows indicate abnormal involuntary movement rating sessions. Closed and open triangles indicate the days of the tracer injection and sacrifice, respectively.

We prepared eight 6-OHDA-lesioned hemiparkinsonian rats (PD model), eight 6-OHDA-lesioned hemiparkinsonian rats with chronic levodopa treatment (LID model), eight control rats with chronic levodopa treatment (levodopa-treated control: LTC model), and nine control rats with saline treatment (Control), as previously described (Ueno et al., 2014; Figure 1).

6-OHDA (8 mg/4 mL in saline with 0.01% ascorbic acid) (Sigma, San Diego, CA, USA) (PD and LID models) or saline (LTC and Control) was injected into the medial forebrain bundle (4.5 mm posterior to bregma, 1.2 mm lateral to the sagittal suture, and 8.5 mm ventral to the dural surface) in the right hemisphere of 10-week-old rats anesthetized with sodium pentobarbital (Nembutal, 50 mg/kg body weight intraperitoneally; Dainippon Sumitomo Pharma Co., Ltd., Osaka, Japan). Apomorphine (Sigma) was administered to evaluate dopaminergic denervation at 12 weeks of age. We previously reported nearly complete dopaminergic denervation in the striatum and M1 with this technique (Maeda et al., 1999; Ueno et al., 2014; Figure 1).

During the 4–6 weeks after the apomorphine test, both 6-OHDA-lesioned rats with dopaminergic denervation and sham-operated rats received 50 mg/kg levodopa methyl ester (Sigma) with 12.5 mg/kg benserazide (Sigma) (LID model and LTC models, respectively) or saline (PD model and Control models, respectively), twice daily (morning and evening) for 14 consecutive days (Figure 1). To evaluate the effects of levodopa, we measured abnormal involuntary movement (AIM) scores (Cenci and Lundblad, 2007) on days 1, 4, and 11 (Figure 1). The AIM score is considered comparable to LID assessments in patients with PD (Cenci and Lundblad, 2007). We observed and scored the rats every 20 min during the 2-h period following levodopa injection. We assessed and summed the scores for the three AIM subtypes (limb, axial, and orolingual) (Cenci and Lundblad, 2007).

### Dendritic spine morphology

We used eight PD models, eight LID models, nine LTC models, and eight controls at 16–18 weeks of age (Figure 1). Our basic method has previously been described in detail (Ueno et al., 2014). To selectively label the cell bodies of PT neurons in the right M1, we stereotactically injected a retrograde tracer, Fast Blue (Polysciences, Inc., Warrington, PA, USA), over a 1-min period into the right pontine pyramidal tract (9.6 mm posterior to bregma, 0.5 mm lateral to the sagittal suture, and 10.7 mm ventral to the dural surface) on day 11 of drug treatment (Paxinos and Watson, 1998; Reiner et al., 2010) (Figure 1). Four days later, the rats were deeply anesthetized with sodium pentobarbital (Nembutal, >75 mg/kg intraperitoneally), intracardially perfused with 4% paraformaldehyde at 12 h after the last levodopa or saline treatment, and the brains then removed.

**Figure 2:**
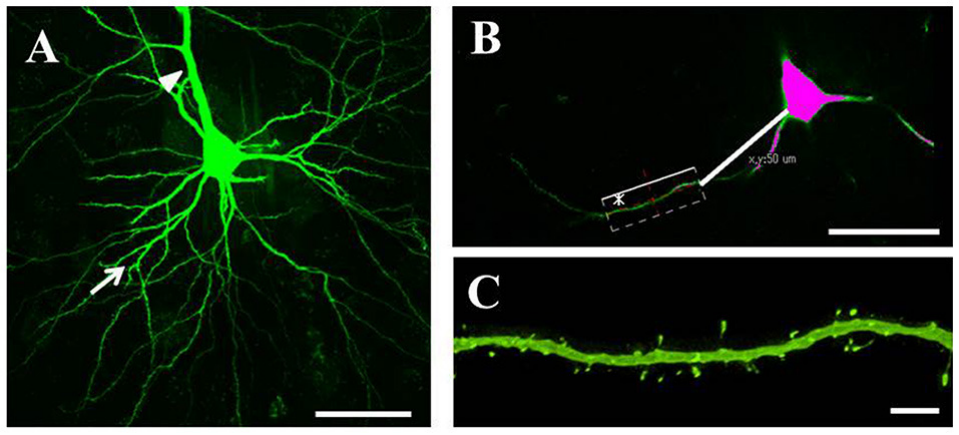
**Analysis of dendritic spines. (A)** Representative confocal images of pyramidal-tract type (PT) neurons in the primary motor cortex. PT neurons were filled with Lucifer Yellow until their dendritic spines became sufficiently visible. We randomly selected several cells for each tissue slice, and several horizontally projecting dendrites for each cell. Arrows: basal dendrites; arrowhead: apical dendrite. Scale bar = 50 μm. **(B)** Dendritic spines were examined at 50–100 μm distal from the cell body. Scale bar = 50 μm. **(C)** Higher magnification view of the area indicated by asterisk in **(B)** showing a basal dendrite 50–100 μm distal from the soma. Small green dots along the basal dendrite represent dendritic spines. Scale bar = 5 μm.

Serial 250-μm-thick coronal sections were cut through the M1, and Lucifer Yellow (Sigma) was injected into cell bodies of Fast Blue-labeled neurons in the right M1 under ultraviolet excitation (380–420 nm) with continuous current (up to 100 nA). Neurons were filled with Lucifer Yellow until their dendritic spines were sufficiently visible (Figure 2A). The tissue was examined by confocal microscopy, and images were taken with a digital camera (C1si; Nikon, Tokyo, Japan). Yellow signals (515/530 nm) were acquired from each sample using 488 nm excitation. Fluorescence projection images of somata and dendritic fields were acquired with a 60 × oil-immersion lens. We selected 5–10 cells for each rat, and 1–5 horizontally projecting dendrites from each cell. We then measured the density and size of spines on the basal dendrite, 50–100 μm distal to the cell body (Figure 2B). Images of the spines in each dendrite were acquired with a 60 × oil-immersion lens (5.0 zoom factor; 0.0064 μm^2^/pixel resolution) at 0.25-μm focal steps. Image stacks were three-dimensional (3D)-deconvoluted using NIS-Elements software (Nikon) and volume rendered as 2D images to facilitate overview of the figures (Figure 2C). In total, we measured 9415 spines from 202 neurons in 33 motor cortices. Each spine was manually traced. The average number of spines per 10 μm of linear dendritic length was expressed as the spine density. All spines were drawn and no distinction was made between different spine types. We measured the cross-sectional area of the spine head in 2D reconstructed images. Image analysis was performed using Image J (National Institutes of Health, Bethesda, MD, USA). For analyses, we selected intracellularly injected cells (Control: 54 cells; PD model: 50 cells; LID model: 49 cells; LTC model: 49 cells) based on our previous criteria (Table 1; Ueno et al., 2014).

**Table 1:** **Number of rat, cell, basal dendrite, and spines analyzed**.

	**Rat**	**Cell**	**Analyzed basal dendrite**	**Analyzed spine**
Control	9	54	162	2,320
Parkinsonian	8	50	152	2,107
Dyskinesia	8	49	153	2,419
Levodopa treated control	8	49	152	2,569

### Statistics

We analyzed the spine density and the average cross-sectional area of the spine heads in each basal dendrite. Statistical analyses were performed with EZR freeware v.1.32 (Saitama Medical Center, Jichi Medical University, Saitama, Japan) (Kanda, 2013). A probability level of 5% (*P* < 0.05) was considered statistically significant. Data are presented as means ± standard error or boxplots showing medians, and 25 and 75% quartile ranges. The spine density, cross-sectional area of the spine heads, and AIM scores were examined using parametric tests (one-way analysis of variance followed by Tukey–Kramer *post-hoc* test), as the Shapiro–Wilk test indicated that the distributions were normal.

## Results

### AIM scores in LID and LTC models

**Figure 3:**
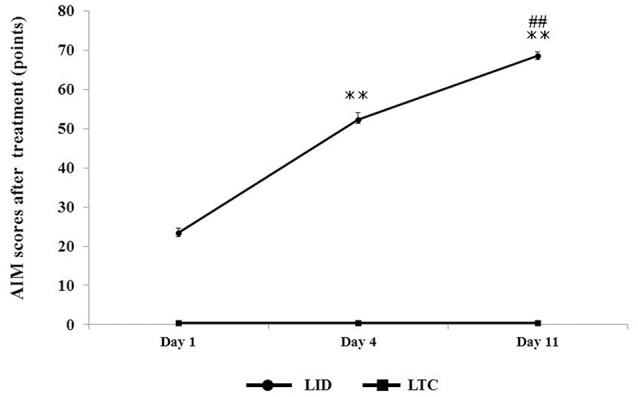
**Abnormal involuntary movement (AIM) scores in LID or LTC groups on days 1, 4, and 11 of treatment**. The scores represent total scores of the three AIM subtypes (limb, axial, and orolingual) (Cenci and Lundblad, 2007). The total score of the three AIMs was significantly increased in the LID group, while no AIMS were observed in the LTC group (one-way analysis of variance followed by *post-hoc* Tukey–Kramer test: ^**^P < 0.001 vs. Day 1, ^*##*^*P* < 0.001 vs. Day 4).

Dopaminergic denervation plus levodopa treatment (LID group) significantly increased AIM scores at day 4 (*P* < 0.001 cf. day 1) and day 11 (*P* < 0.001 cf. day 4), whereas levodopa treatment had no effect on AIM scores in control rats (LTC group) (Figure 3).

### Morphological changes in dendritic spines of PT neurons

**Figure 4:**
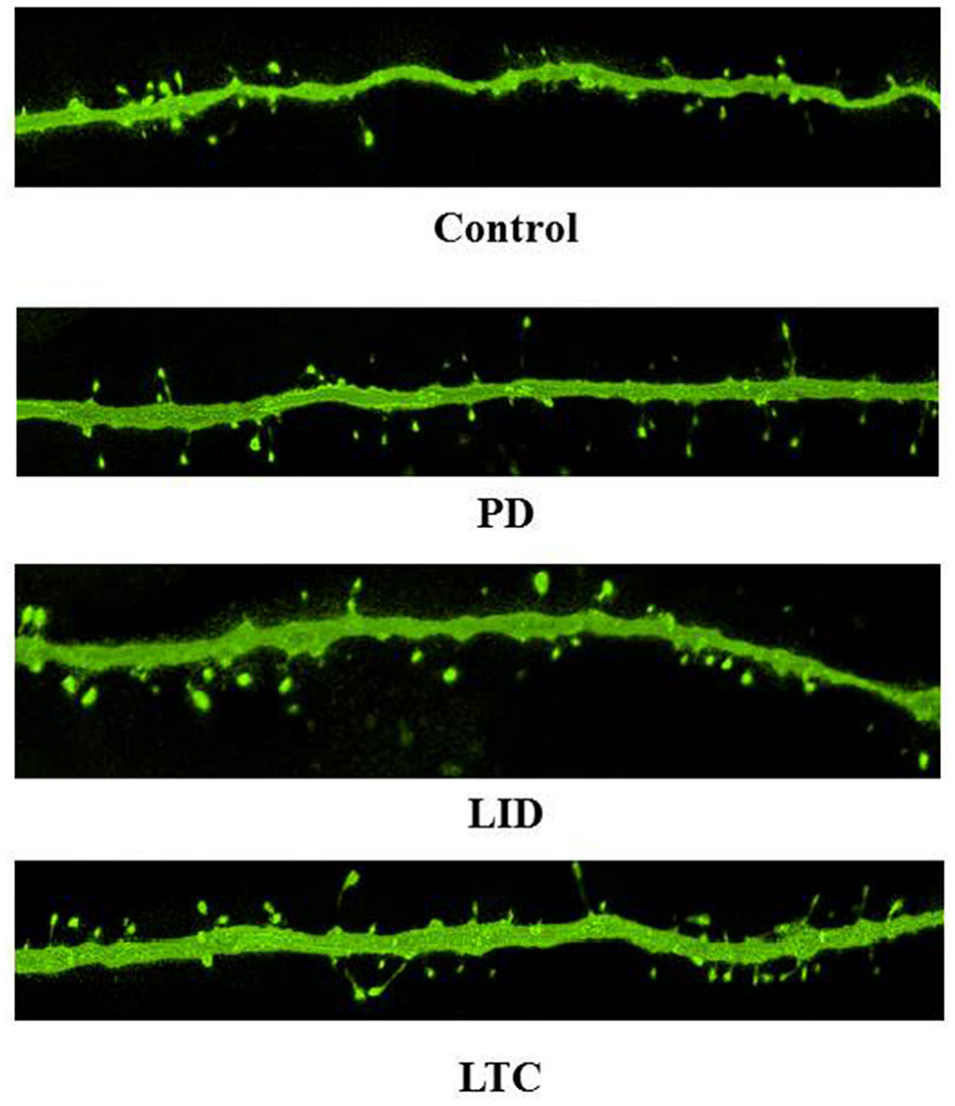
**Representative confocal microscopy images of dendritic spines on PT neurons. Scale bar = 5 μm**.

**Figure 5:**
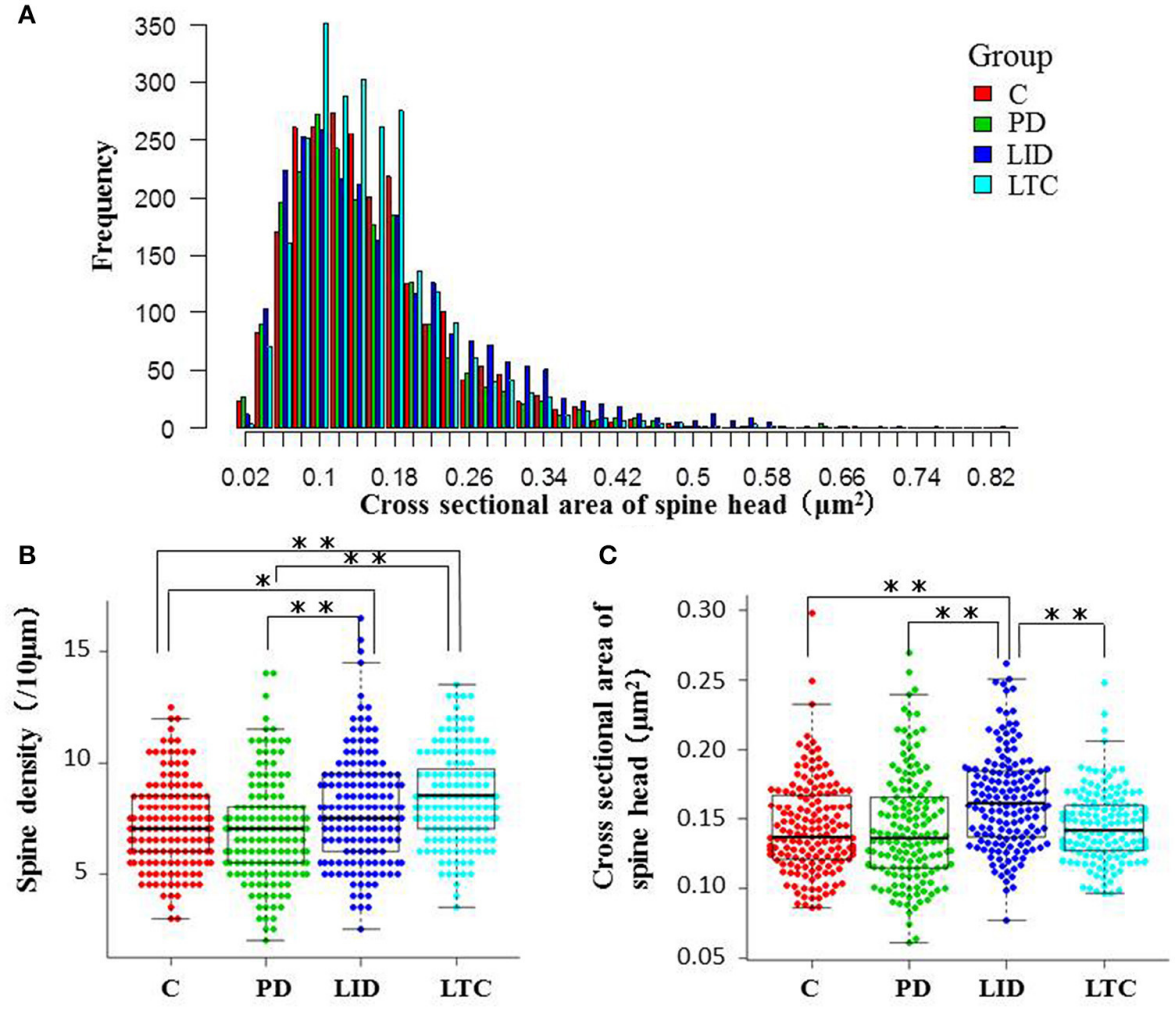
**Morphological evaluation of spines on basal dendrites. (A)** Histograms of cross-sectional areas of the analyzed spine heads. The red, green, blue, and light blue bars indicate control, PD, LID, and LTC groups, respectively. The histogram shows that the cross-sectional area of the spine heads was increased in the LID group compared with the other groups. **(B)** Spine density was significantly increased in the LID and LTC groups compared with the Control and PD groups. **(C)** Spine size was significantly increased in the LID group (^*^P < 0.05, ^**^P < 0.001, one-way analysis of variance followed by *post-hoc* Tukey–Kramer test). C, control.

Forty-five animals underwent histological examinations with 12 excluded due to unsatisfactory histology. We analyzed the spine density and average cross-sectional area of spine heads in 619 basal dendrites (control = 162, PD = 152, LID = 153, LTC = 152) (Table 1; Figure 4). Using histograms from 9,415 cross-sectional areas of spine heads, the LID group showed significantly enlarged spine heads compared with the other groups (*P* < 0.001) (Figure 5A).

Levodopa treatment of the dopaminergic denervation (LID group) and control rats (LTC group) significantly increased the spine density of M1 PT neurons compared with the Control (*P* < 0.05 cf. LID; *P* < 0.001 cf. LTC) and PD groups (*P* < 0.001 cf. LID; *P* < 0.001 cf. LTC) (Control group: 7.2 ± 0.15/10 μm; PD group: 7.0 ± 0.20/10 μm; LID group: 7.9 ± 0.20/10 μm; LTC group: 8.5 ± 0.17/10 μm). However, dopaminergic denervation (PD group) had no effect on spine density of PT neurons compared with the Control group. No significant differences were observed between the LID group and the LTC group (Figures 4, 5B).

Dopaminergic denervation (PD group) had no effect on the spine size of PT neurons, while dopaminergic denervation plus levodopa treatment (LID group) significantly enlarged dendritic spines compared with the Control group, the PD group, and the LTC group (*P* < 0.001) (Figures 4, 5C). However, levodopa treatment of control rats (LTC group) had no effects on spine size (Control group: 0.14 ± 0.003 μm^2^; PD group: 0.14 ± 0.003 μm^2^; LID group: 0.16 ± 0.003 μm^2^; LTC group: 0.14 ± 0.002 μm^2^) (Figures 4, 5C).

## Discussion

In this study, we demonstrated that chronic levodopa treatment in normal and LID model rats increases the spine density of PT neurons in the M1. The dendritic spines of M1 PT neurons became enlarged in the LID model, and this enlargement of spines appears to be relevant to the development of AIMs. This structural change suggests that PT neurons become supersensitive to glutamatergic inputs in dyskinetic rats.

### Effect of dopaminergic denervation on dendritic spines of M1 PT neurons

We found that dopaminergic denervation alone had no effect on M1 PT neuron spine density or size (Figures 4, 5B,C). The preservation of spine density in the motor cortex after dopaminergic denervation is comparable with previous studies (Miklyaeva et al., 2007; Wang and Deutch, 2008; Ueno et al., 2014). However, Guo et al. (2015) recently reported that both spine elimination and formation of layer V pyramidal neurons of the primary motor cortex were increased in a mouse model of PD induced by 1-methyl-4-phenyl-1,2,3,6-tetrahydropyridine, resulting in a decrease in spine density in a PD mouse model. Although, the exact causes of the differences in spine density between these studies remain unclear, they may relate to whether basal or apical dendrites were analyzed. For example, no significant changes in spine density were found in three studies examining the basal dendrites of layer V pyramidal neurons (Miklyaeva et al., 2007; Wang and Deutch, 2008; Ueno et al., 2014). By contrast, Guo et al. (2015) demonstrated a decrease in the spine density of apical dendrites of layer V pyramidal neurons; a similar, but not significant, trend has also been reported (Wang and Deutch, 2008).

There were also differences between these studies in the timing of measurements after dopaminergic lesioning (Miklyaeva et al., 2007; Wang and Deutch, 2008; Ueno et al., 2014; Guo et al., 2015). Differences in the methodological approaches to visualizing spines may also be important. Although, previous studies have used the Golgi-Cox method (Miklyaeva et al., 2007; Wang and Deutch, 2008) and confocal laser scanning microscopy (Ueno et al., 2014), Guo et al. (2015) used two-photon laser scanning microscopy. The cell types examined may also result in differences. In studies other than ours, spine morphology was examined without discriminating between PT and IT neurons (Miklyaeva et al., 2007; Wang and Deutch, 2008; Ueno et al., 2014; Guo et al., 2015). As IT and PT neurons express D1 and D2 dopamine receptors, respectively, (Gee et al., 2012; Seong and Carter, 2012; Dembrow and Johnston, 2014), dopaminergic denervation may differentially impact the spine density of these cell types. Finally, differences in the methods used to induce dopaminergic denervation (e.g., local application of 6-OHDA into dopamine neurons versus generalized administration of 1-methyl-4-phenyl-1,2,3,6-tetrahydropyridine) may be an important factor contributing to these contrasting findings.

### Increased spine density of M1 PT neurons with chronic levodopa treatment with or without dopaminergic denervation

Here we showed that chronic levodopa treatment with or without dopaminergic denervation increases the spine density of PT neurons in M1 (Figures 4, 5B). Levodopa normalizes the increase in spine turnover in the M1 following dopaminergic denervation (Guo et al., 2015). Thus, levodopa treatment may affect spine turnover in both the dopamine-denervated M1 and the dopamine-intact M1. However, we previously reported that chronic levodopa treatment in PD model and control rats does not change the spine density of M1 IT neurons (Ueno et al., 2014). The distribution of dopamine receptors may underlie these differences in spine density between IT and PT neurons. Although, the specific distribution of dopamine receptors in layer V pyramidal neurons of the M1 remains to be determined, IT and PT neurons in the mouse medial prefrontal cortex express D1 and D2 receptors, respectively (Gee et al., 2012; Seong and Carter, 2012; Dembrow and Johnston, 2014). D1 dopamine receptor signaling regulates spine elimination in the M1, while D2 receptor signaling controls spine formation in the M1 (Guo et al., 2015). Taken together, the differences in the effects of chronic levodopa treatment in naive rats may be dependent on the dopamine receptor type. Levodopa treatment increases dopamine levels in the motor cortex without dopaminergic denervation (Navailles et al., 2011). Thus, the increase in PT neuron spine density may be induced by the D2 receptor response in the M1. Different analysis methods may also contribute to the differences in spine density in our two studies: the spine density of each dendrite was measured in the present study, while we previously used the average spine density of each neuron, and then the average for each rat (Ueno et al., 2014).

### Enlargement of dendritic spines of M1 PT neurons of LID model rats

Dopaminergic denervation or levodopa treatment alone had no effect on dendritic spine size (Figures 4, 5C). However, levodopa treatment after dopaminergic denervation enlarged dendritic spines in PT neurons, with the appearance of dyskinetic movements (Figures 3, 4, 5C). Thus, chronic levodopa treatment after dopaminergic denervation results in the enlargement of dendritic spines in both IT (Ueno et al., 2014) and PT neurons. This structural change suggests that PT neurons also acquire supersensitivity to glutamatergic inputs in dyskinesia-primed rats, as is the case for IT neurons in the M1 (Ueno et al., 2014).

We previously reported that dendritic spines become enlarged in both dSPNs in the striatum and IT neurons in the M1 cortex of the same LID model (Nishijima et al., 2014; Ueno et al., 2014). Loss of depotentiation after induction of LTP at corticostriatal synapses is a key neurophysiological feature of LID models (Picconi et al., 2003; Shen et al., 2015), and a similar loss of depotentiation-like plasticity has been demonstrated in PD patients using transcranial magnetic stimulation (Huang et al., 2011). Dendritic spines form the postsynaptic compartment of the majority of excitatory glutamatergic synapses in the brain (Murakoshi and Yasuda, 2012). Dendritic spine size is tightly correlated with synaptic strength (Matsuzaki et al., 2001), and is actively regulated during synaptic plasticity (Matsuzaki et al., 2004). Spines display long-lasting enlargement during LTP (Matsuzaki et al., 2004; Harvey and Svoboda, 2007), which results from actin polymerization and insertion of AMPA receptors into the dendritic spines (Matsuzaki et al., 2004; Rudy, 2015). These events lead to an increase in the sensitivity of postsynaptic sites to glutamate (Murakoshi and Yasuda, 2012). As enlargement of dendritic spines indicates a supersensitivity of excitatory synapses (Segal, 2010), our data support a lack of potentiation in the motor cortex in LID (Huang et al., 2011). Taken together, the loss of depotentiation may correlate with enlargement of dendritic spines in dyskinesia-primed animal models. The supersensitivity of the synapses of cortical motor neurons may contribute to the strengthened signal transduction demonstrated in LID models (Ren et al., 2011; Yang et al., 2012a,b; Xie et al., 2014). Accordingly, the expression of LID is inhibited by reduction of cAMP-dependent protein kinase, dopamine and cAMP-dependent phosphoprotein of 32 kDa, and phosphorylated glutamate receptor 1 (Ren et al., 2011; Yang et al., 2012a,b; Xie et al., 2014). These molecules are essential for the activation of Ca2+/calmodulin–dependent protein kinase II, which is associated with the enlargement of dendritic spines (Yagishita et al., 2014).

Although, we did not measure the synaptic function of PT neurons using electrophysiology in this study, the spine enlargement of PT neurons probably results in supersensitivity of PT neurons to glutamatergic input in the M1 (Ueno et al., 2014), and may underlie the emergence of LID. IT-type inputs to dSPNs and PT-type inputs to iSPNs may show short-term facilitation, whereas IT-type inputs to iSPNs and PT-type inputs to dSPNs may show short-term depression (Morita, 2014). IT-dSPN and PT-iSPN synapses evoke short-term facilitation, whereas IT-iSPN and PT-dSPN synapses evoke depression (Shipp, 2017). Thus, IT and PT neurons have complementary effects on dSPNs and iSPNs. The enlargement of IT neuron spines in our previous study (Ueno et al., 2014), and of PT neuron spines in the present study, indicates the enhanced sensitivity of pyramidal neurons in the M1. This may relate to the generation of abnormal oscillation in the cortex of dyskinetic animal models (Halje et al., 2012; Dupre et al., 2016). Here, we provide further evidence for the storage of abnormal plastic information in the M1 following dyskinetic movements.

## Conclusions

In the primary motor cortex, chronic levodopa treatment modifies the formation of dendritic spines in PT neurons with or without dopaminergic denervation. Furthermore, chronic levodopa treatment after dopaminergic denervation causes the enlargement of PT neuron dendritic spines. These results suggest that spine enlargement in PT neurons may be a key factor in the development of LID in the M1.

## Author contributions

Conception and design of the study, data interpretation, drafting the article or revising it critically for important intellectual content, and final approval of the version to be submitted: TU, HN, SU, and MT; Data acquisition and analysis: TU.

## Funding

This study was supported by a grant-in-aid for scientific research from the Ministry of Education, Culture, Sports, Science and Technology of Japan (No. 22590952) to MT. The funding source had no role in study design, data collection, data analysis, data interpretation, or writing of the report.

### Conflict of interest statement

The authors declare that the research was conducted in the absence of any commercial or financial relationships that could be construed as a potential conflict of interest.

## Abbreviations

3D: 3-dimensional
6-OHDA: 6-hydroxydopamine
AIM: abnormal involuntary movement
dSPN: direct pathway striatal projection neurons
iSPN: indirect pathway striatal projection neurons
IT: intratelencephalic
LID: levodopa-induced dyskinesia
LTC: levodopa-treated control
LTP: long-term potentiation
M1: primary motor cortex
PD: Parkinson's disease
PT: pyramidal tract.
